# 12p13.2杂合缺失综合征一例：先天性血小板减少、缺指和牙齿畸形

**DOI:** 10.3760/cma.j.issn.0253-2727.2021.11.014

**Published:** 2021-11

**Authors:** 圆 徐, 云飞 陈, 荣凤 付, 葳 刘, 满凯 鞠, 婷 孙, 慧媛 李, 峰 薛, 磊 张, 仁池 杨, 晓帆 刘

**Affiliations:** 中国医学科学院血液病医院（中国医学科学院血液学研究所），实验血液学国家重点实验室，国家血液系统疾病临床医学研究中心，天津 300020 State Key Laboratory of Experimental Hematology, National Clinical Research Center for Blood Diseases, Institute of Hematology & Blood Diseases Hospital, Chinese Academy of Medical Sciences & Peking Union Medical College, Tianjin 300020, China

患者，女，24岁，因“发现血小板减少6年”于2020年12月18日来我院门诊就诊。患者6年前因感冒至当地医院查血常规示血小板21×10^9^/L，白细胞、红细胞无异常，至当地医院就诊，查血常规示WBC 7.05×10^9^/L、HGB 154g/L、PLT 22×10^9^/L。骨髓象：增生活跃，退化细胞可见，粒系占55.2％，少数中幼粒细胞有核浆发育失衡现象，红系占27.2％，中幼红比例增高，可见核分裂象，成熟红细胞大小不均，淋巴比例减低，可见异型淋巴细胞，全片见巨核183个，分类20个，其中成熟无血小板形成巨核细胞15个，成熟有血小板形成巨核细胞3个，裸核巨核细胞2个，血小板少见，意见：巨核细胞增多伴成熟障碍；骨髓活检：巨核细胞轻度增多，给予泼尼松40 mg/d，1周后复查血小板计数升至101×10^9^/L，后泼尼松逐渐减量，约2年激素减停，期间血小板计数最低60×10^9^/L。后口服中药治疗4年，多次复查血小板计数（30～60）×10^9^/L。2020年12月2日至当地医院就诊，查血常规示PLT 29×10^9^/L，骨髓象：增生活跃，粒系62％，未见发育异常，红系17％，全片见巨核细胞27个，其中成熟无血小板形成巨核细胞17个，成熟有血小板形成巨核细胞1个，裸核巨核细胞9个，血小板少见。给予重组人血小板生成素、升血小板胶囊、脾多肽等治疗2周，复查PLT 62×10^9^/L，遂转诊于我院。患者既往有“小脑发育不全”病史。查体：右手缺一指，牙齿稀少，排列不齐，发育缺陷，头发稀疏。血常规：WBC 7.28×10^9^/L、HGB 131 g/L、PLT 63×10^9^/L、RBC 5.1×10^12^/L、网织红细胞1.36％、中性粒细胞绝对计数4.26×10^9^/L；铁蛋白7.20 µg/L；D-二聚体1.23 mg/L；生化全套：丙氨酸氨基转移酶39.60 U/L，甘油三酯1.99 mmol/L；血清铁7.61 µmol/L，未饱和铁61.76 µmol/L，铁饱和度0.11；肿瘤标记物等其他指标未见异常。外周血全外显子二代检测（由北京迈基诺基因检测公司完成）：12p13.2p13.1区域存在杂合缺失chr12:10337873-14664645（12号染色体短臂存在大小约4.326 Mb的缺失，该区域内包含剂量敏感基因CDKN1B、ETV6、GRIN2B和LRP6），患者父亲、母亲chr12:10337873-14664645区域均无缺失。基于患者临床表现与测序结果，诊断为12p13.2杂合缺失综合征。

讨论：12p13.2p13.1区域涉及到多种基因，故该区域杂合缺失的患者可能会出现多种临床症状。

本例患者为青年女性，表现为血小板减少、右手缺指和牙齿畸形等多系统损害，二代测序检出12p13.2p13.1区域存在杂合缺失，该区域内包括ETV6、LRP6、GRIN2B和CDKN1B基因。根据美国医学遗传学与基因组学会（ACMG）发布的变异解读指南，上述变异初步判定为致病性变异。ETV6、LRP6、GRIN2B和CDKN1B基因分别与遗传性血小板减少症/血液系统肿瘤、选择性牙齿/手指发育不全、常染色体显性智力低下/发育性癫痫性脑病、多发性内分泌腺瘤的发病密切相关。本例患者无遗传病家族史，迄今无明显出血倾和肿瘤转化表现。

